# Improving the Efficiency of Respiratory Drug Delivery: A Review of Current Treatment Trends and Future Strategies for Asthma and Chronic Obstructive Pulmonary Disease

**DOI:** 10.1007/s41030-017-0046-2

**Published:** 2017-06-27

**Authors:** Ayah Shakshuki, Remigius U. Agu

**Affiliations:** Biopharmaceutics and Drug Delivery Lab, College of Pharmacy, Halifax, Canada

**Keywords:** Asthma, Chronic obstructive pulmonary disease (COPD), Adherence, Inhaler development, Future treatments

## Abstract

Asthma and chronic obstructive pulmonary disease (COPD) are heterogeneous airway diseases associated with significant morbidity and mortality. Pharmacological treatment is delivered primarily through the inhalation route using various devices. Optimal disease control is highly dependent upon patient adherence. Both patients with asthma and COPD are prone to exacerbations leading to hospitalization, which can significantly impact quality of life. Poor adherence is a complex and multifactorial problem that does not have one simple solution. However, it is the biggest risk factor for exacerbations and consequently high healthcare utilization. This review discusses the complex and multifactorial obstacles that impact patient adherence as well as the effect on overall treatment outcomes and healthcare utilization. We also critically examined and compared relatively recent improvements in breath-activated pressurized metered dose inhalers, dry powder inhalers, and e-technology in asthma and COPD. Finally, future treatment strategies for better patient compliance such as personalized medicine and the importance of decision-making between patients and physicians were highlighted.

## Background to Treatment of Asthma and COPD

### Asthma

Asthma is a common and chronic airway disease which affects approximately 300 million adults and children worldwide [1]. It is characterized by chronic airway inflammation and increased bronchial hyerresponsiveness that can lead to a variety of symptoms typically seen in asthmatic patients such as cough, wheeze, chest tightness, and shortness of breath [2, 3]. These symptoms often occur after exposure to viral infections, exercise, cold air, allergens, strong smells, and a plethora of environmental irritants [4]. Patients have variable airway obstruction that is often reversible by using inhaled medication [5]. However, with prolonged suboptimal treatment or no treatment at all, airway remodeling may begin to occur and result in airway obstruction that is only partially reversible [2].

Once the diagnosis of asthma is confirmed by spirometry, determining disease severity based on symptoms, nighttime awakening, and interference with normal activity aids in guiding initial treatment [6]. The National Asthma Education and Prevention Program (NAEPP) uses the Rules of Two^®^ to differentiate patients who have intermittent asthma and persistent asthma [7]. Patients with intermittent asthma have symptoms less than 2 days a week, nighttime awakening less than two nights a month, and require a short-acting β_2_-agonist (SABA) less than 2 days a week, have normal spirometry results between exacerbations, and have no disruptions in daily activities due to their asthma [6]. In contrast, patients with persistent asthma have symptoms much more frequently and are stratified into mild, moderate, and severe asthma on the basis of frequency of symptoms, nighttime awakenings, use of a SABA, lung function, and interference with daily activities [6].

**Fig. 1:**
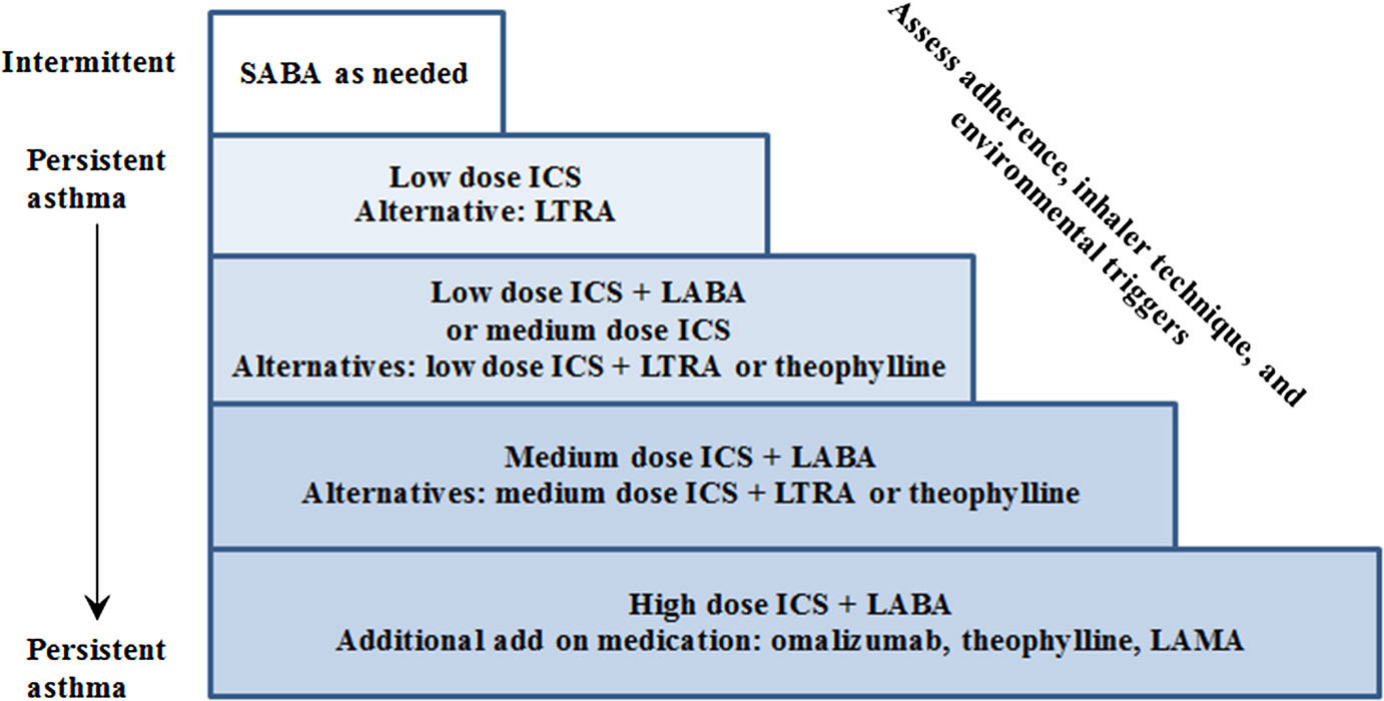
Stepwise approach to treatment of asthma. *SABA* short-acting β_2_-agonist, *ICS* inhaled corticosteroid, *LTRA* leukotriene receptor antagonist, *LABA* inhaled long-acting β_2_-agonist, *LAMA* short-acting antimuscarinic antagonist

On the basis of NAEPP’s classification, those with intermittent asthma only require a SABA without any regular maintenance treatment; however, there is emerging evidence towards regular inhaled corticosteroid (ICS) use as it reduces decline in lung function and asthma-related hospitalizations [6, 8, 9]. Figure 1 summarizes a stepwise approach for the treatment of asthma. Should asthma control not be achieved with a certain level of treatment, inhaler technique, patient adherence, environmental triggers, and patient comorbidities (e.g., allergic rhinitis) should be explored as possible contributing factors. Patients with persistent symptoms and exacerbations, despite correct inhaler technique and good treatment adherence, should be referred to a specialist who has experience in dealing with this patient population [4]. At this point, other options such as tiotropium (a long-acting antimuscarinic), oral corticosteroids, and omalizumab (anti-immunoglobulin E) may be considered [4, 6, 10].

Asthma severity is not static in nature and should be reassessed once the patient has been on maintenance medication for 3–4 months [6]. As with any disease state, it is important to find the minimum effective level of treatment offering the least amount of side effects to the patient. As such, if a patient has stable lung function and well-controlled symptoms for at least 3 months with no major risk factors for exacerbations, Global Initiative for Asthma (GINA) advocates stepping down treatment [4]. Treatment goals of asthma include preventing mortality due to asthma, improving quality of life by allowing for maintenance of normal activities (school, work, exercise, etc.), reducing daytime and nighttime symptoms of cough, wheeze, and shortness of breath, preventing acute asthma exacerbations, and providing treatment with minimal side effects [4, 9]. This article is based on previously conducted studies and does not involve any new studies of human or animal subjects performed by any of the authors.

### Chronic Obstructive Pulmonary Disease

Chronic obstructive pulmonary disease (COPD) is also a common airway disease affecting 210 million adults globally and is a cause of significant morbidity and mortality [5]. It is a progressive disease that results in airflow limitation due persistent inflammation in the airways, mucociliary dysfunction, lung hyperinflation, and destruction of the lung parenchyma [11, 12]. Although long-term exposure to inhaled irritants, such as cigarette smoke, is the most common cause of COPD, breathing in chemical fumes, air pollution, alpha-1 antitrypsin deficiency, and persistent untreated asthma can also lead to the development of this disease [13]. The impact of COPD on a patient’s quality of life is dependent on exercise capacity, comorbidities, and the severity of symptoms such as dyspnea, cough, phlegm production, and chest tightness [5, 12]. Additionally, COPD is associated with systemic or extrapulmonary effects such as right heart failure, skeletal muscle dysfunction, osteoporosis, and weight loss [11]. The goal of treatment is to reduce symptoms, improve exercise tolerance, improve quality of life, prevent exacerbations, and reduce mortality [12].

One of the biggest differences between asthma and COPD is that airway obstruction is not fully reversible after using a bronchodilator [5]. An FEV_1_/FVC ratio of less than 0.7 after using a bronchodilator is one of the diagnostic criteria for COPD [14]. FEV_1_ by itself does not correlate well with patient symptoms, impairment, disability, and risk of mortality and should not be used as the sole manner of categorizing severity and guiding therapy [12, 14]. Unfortunately, to obtain drug coverage for long-acting muscarinic antagonist (LAMA) and other inhalers in certain provinces in Canada, severity is primarily based on FEV_1_ by the government drug plan. FEV_1_ ≥ 80% predicted, 50% ≤ FEV_1_ < 80% predicted, 30% ≤ FEV_1_ < 50% predicted, and FEV_1_ < 30% predicted are considered to be mild, moderate, severe, and very severe lung impairment, respectively [1, 14]. The modified Medical Research Counsel dyspnea scale (mMRC), the COPD assessment test (CAT™), and the BODE index are a few of many grading systems that exist for COPD severity categorization, with no consensus on which system is superior [12, 14].

**Fig. 2:**
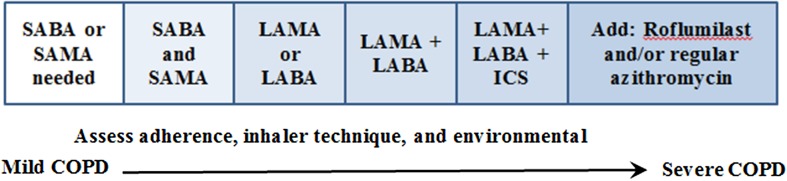
*SABA* short-acting β_2_-agonist, *SAMA* short-acting antimuscarinic antagonist, *LABA* long-acting β_2_-agonist, *LAMA* short acting antimuscarinic antagonist, *ICS* inhaled corticosteroid

Maintaining optimal bronchodilation is important to improve patient symptoms as well as reduce mortality [15]. In addition to pharmacotherapy, which is summarized in Fig. 2, smoking cessation initiated early in the disease can slow disease progression and improve symptoms [14]. There is sparse evidence on whether a LAMA or LABA is superior when a long-acting agent is required; however, there is some data indicating that the LAMA tiotropium may be associated with a greater reduction in exacerbations and hospitalizations compared with the LABA salmeterol [12, 16]. If COPD is severe with frequent exacerbations, an ICS may be added; however, use of an ICS has been found to increase the risk of pneumonia [17]. If inadequate symptom relief and reduction in exacerbations are observed despite combination pharmacotherapy with correct inhaler technique, roflumilast, a phosphodiesterase-4 inhibitor, and/or adding the antibiotic azithromycin may be considered by a specialist [12, 14].

## Adherence and Consequences for Patients’ Asthma and COPD

### Factors that Impact Patient Adherence

For inhaled medications to deposit in the airways and be effective, patients must use them regularly with the correct technique [18]. This is especially important to reduce the likelihood of negative outcomes in asthma and COPD including poorer quality of life, reduced productivity, increased hospitalization, worsening of disease, and increase in mortality risk [1, 4, 12]. Medication adherence is defined as how closely a patient follows the recommended treatment plan mutually agreed upon with their health care provider [19]. Nonadherence can be either intentional or unintentional with intentional nonadherence involving patients actively choosing not to follow treatment prescribed for a variety of reasons [19]. Unintentional nonadherence often involves patients wanting to use the treatment prescribed, but either do not use it at all, or deviate from the directions given as a result of misunderstanding instructions, forgetfulness, lack of affordability, and poor inhaler technique [20]. The term adherence is preferred over the term compliance, as compliance implies the patient is to follow the recommendation of their healthcare provider(s) without question, which goes against the movement towards patient-centered healthcare. Nonadherence, both intentional and unintentional, is important to frequently assess as it can not only change during the treatment course but can also be mistakenly labeled as refractory illness [20].

It is estimated that medication adherence rates for patients with asthma and COPD are less than 50%, which is quite low considering both the patient and economic consequences of poor disease control [21–23]. Nonadherence in COPD and asthma includes patients not initiating inhaler treatment, using less than prescribed, using more medication than prescribed, using the inhaler device in an inappropriate manner, and stopping treatment prematurely [19]. To devise individualized plans to tackle this problem, it is important to recognize that the reasons driving patient nonadherence are often multifactorial. Reasons for poor adherence, as seen in Table 1, can be broadly categorized into patient-, healthcare provider-, and healthcare system-related factors [24]. A patient-related factor may include having a lack of understanding of the disease itself and the progression and consequences of not using any form of treatment [24]. It is difficult to be adherent to any form of treatment for any chronic disease if a person does not understand why they were prescribed the medication in the first place, particularly if the benefits of treatment are not immediately apparent. An example of this is the use of ICS as maintenance treatment in asthma, where it takes weeks to months to have a noticeable benefit [4]. In contrast, a SABA provides immediate relief of symptoms and its use can easily be justified by a patient. Other patient factors include age, low socioeconomic status, lack of transportation to the pharmacy, personal beliefs and fears around using medications, patient comorbidities, cognitive and functional impairment, and family support [25]. Healthcare provider factors that can significantly impact patient adherence include prescribing complex medication regimens, not considering affordability of medications, lack of counseling on the disease as well as the risk versus benefit of treatment, not thoroughly demonstrating inhaler technique, and overall ineffective communication between provider and patient [24, 25]. Although both patient factors and healthcare provider factors are important to address, issues with the healthcare system itself, which is beyond the control of the individual, can create barriers [24]. Healthcare system factors include high drug costs, poor communication between specialists, hospitals, and primary care physicians, high patient load on family physicians, patient lack of access to a family physician, and lack of time for a clinician to properly counsel and assess patient adherence [24].

**Table 1:** Factors that can impact patient adherence

Patient factors	Health care provider factors	Health care system factors
Age Socioeconomic status Transportation Fear of medication Values and beliefs Comorbidities Cognitive function Poor understanding of disease Family support	Complicated treatments Disregarding medication price Patient education Inhaler technique Communication barriers	Poor communication between specialist, hospital, and primary physician High physician patient load Lack of patient access to a family physician Lack of clinician time to properly counsel and assess patient

### Effect of Patient Adherence on Treatment Outcomes and Healthcare Utilization

Adherence with prescribed therapy is generally associated with better symptom control and improvement in lung function in asthmatics [22]. However, nonadherence can lead to poor disease control, which, if not recognized as the reason for symptoms, can be mistakenly diagnosed as refractory illness and lead to inappropriately escalating medication doses and addition/changes in therapy [20]. Additionally, both asthma and COPD symptoms can have a significant impact on school, work, physical activities, and social life [1]. There is a clear trend of increased hospitalizations, mortality, loss of productivity as well as overall poorer quality of life in nonadherent patients [26]. Using disability-adjusted life years, the World Health Organization (WHO) ranks COPD 10th and asthma 22nd in terms of diseases causing the greatest burden globally [1]. Amongst chronic diseases, COPD is the fifth leading cause of death, with treatment found to significantly reduce mortality [1].

Both the direct and indirect economic costs of asthma and COPD are quite considerable [22]. Direct costs include hospitalization, nursing care, physician services, and prescription costs [27]. Indirect costs include poorer work performance, missed days of work, lost wages, employer costs, lost wages of caregivers, and disability [27]. In Canada, approximately 70,000 emergency department visits a year are due to asthma with the direct and indirect costs being greater than CAD $2.1 billion [28]. In the USA, total annual costs of COPD are estimated to be $52.4 billion, with hospitalizations comprising the bulk of this [12]. Exacerbations leading to hospitalization or emergency department use are the main source of healthcare costs [22]. Therefore, appropriate persistent treatment as well as good patient adherence is expected to reduce these costs as they are associated with decreased exacerbations.

## Recent Improvement in Inhaler Development

Inhalation devices used in asthma and COPD include nebulizers, pressurized metered dose inhalers (pMDIs), soft-mist inhalers, and dry powder inhalers (DPI) [12]. The most important factors that determine drug deposition by inhalation are the inhaled particle size, aerosol velocity, and patient inspiratory flow [29]. Particle size is described as either mass mean aerodynamic diameter (MMAD) or fine particle fraction (FPF) [18]. FPF is defined as the proportion of particles which are less than 5 microns in diameter [18]. Drug particles greater than 5 microns are most likely to be deposited in the oropharynx, while particles between 2 and 5 microns have the greatest likelihood of being deposited throughout the bronchial tree [29]. Although pMDIs are one of the most widely used inhalation devices, they require adequate coordination between device actuation and inhalation as well as breathing slowly and deeply, which can be challenging for patients [29, 30]. It is not unusual for patients to actuate the device before inhaling, which leads to less drug deposition in the airways and hence a suboptimal clinical effect [31, 32]. To eliminate the coordination issue associated with pMDIs, research and development around breath-activated inhalers has greatly increased in the last decade [32]. Breath-activated inhalers can be in the form of a pMDI or a DPI.

### Breath-Activated Pressurized Metered Dose Inhalers

Breath-activated pressurized metered dose inhalers (BA-pMDIs) contain the same pressurized canister as a pMDI, but instead of coordinating inhalation with actuation of the device, they contain a triggering mechanism which releases the dose after detecting a patient’s inspiration [31, 33]. The Autohaler^®^ and the Easi-Breathe^®^ are the only two commercial BA-pMDIs that are available [18]. No such product exists on the Canadian market. The Autohaler^®^ requires inspiratory rates of 30 L/min while Easi-Breathe^®^ requires rates of 20 L/min [18]. These inspiratory flow rates are readily attainable by most patients [32]. In contrast, traditional pMDIs require inspiratory rates between 30 and 60 L/min [33]. Both BA-pMDIs are believed to deliver an equivalent dose to the airways as a pMDI used with good technique [34]. However, in those who actuate a beclomethasone pMDI prior to inhaling, the mean drug deposition in the lung is 23% less than if using a BA-pMDI [35]. Other BA-MDIs that do not have any clinically used commercial products available include the K-Haler^®^ and the MD Turbo^®^ [36].

### Dry Powder Inhalers

Dry powder inhalers are all breath actuated and therefore do not have the issue of coordinating actuation and inhalation [18]. There has been a greater shift in research and development to DPIs partly due to their enhanced drug formulation stability, flexibility in inhaler design options, incorporation of hydrophobic drugs, and ability to achieve a high FPF [37, 38]. DPIs are categorized as either passive or active depending on whether they are dependent on patient inspiratory flow [39].

For lung deposition to occur with passive inhalers, turbulent energy, which is formed as a product of patient inspiratory flow as well as the DPI device’s internal resistance, de-agglomerates drug particles to an appropriate size [18, 40]. The minimal turbulent energy required for particles to reach the optimal MMAD varies for each DPI device [18]. A higher inspiratory rate is expected to result in a higher rate of particle de-agglomeration [40]. The downside of this is that a higher inspiratory rate also results in increased drug particle velocity, which is expected to lead to higher oropharyngeal drug deposition [41]. Additionally, a high inspiratory rate may be difficult to attain in children, elderly, and in certain patients with COPD and asthma [42]. A device with a generated peak inspiratory rate of 90, 60–90, 50–60, and less than 50 L/min is categorized as low, medium, medium–high, and high resistance inhaler, respectively [18]. The intrinsic resistance refers to the inspiratory flow rate required to produce a decrease in pressure of 4 kPa in the device [18]. There appear to be many misunderstandings in the literature regarding inspiratory flow rates and inspiratory effort [43]. It is expected that patients using DPIs will exert maximal inspiratory effort to generate a certain inspiratory airflow in the device [44]. This inspiratory airflow will vary depending on the resistance of the device. Therefore, to achieve the same inspiratory flow, patients using a high resistance inhaler would need to have a greater inspiratory effort than a low resistance device [44]. Unlike older devices on the market, DPIs approved in the past 5 years have shown FPF greater than 20% and require a lower inspiratory rate [45]. Four inhaler devices approved in the past 5 years include Genuair^®^, Ellipta^®^, Nexthaler^®^, and Breezhaler^®^ (Table 2).

**Table 2:** Summary of relatively new dry powder inhalers

Device	Drugs	Intrinsic resistance	Inspiratory flow rate (L/min)
Genuair^®^	Aclidinium bromide	Medium	64
Ellipta^®^	Fluticasone furoate and vilanterol	Medium	74
Nexthaler^®^	Formoterol fumarate and beclomethasone dipropionate	Medium–high	54
Breezhaler^®^	Indacaterol and glycopyrronium bromide	Low	90–100

Genuair^®^, which was approved by the FDA in 2012 for the treatment of COPD, is a multidose DPI with the long-acting antimuscarinic aclidinium bromide [42]. Genuair^®^ is also known as Novolizer^®^ or Pressair^®^ in different countries [46]. The device is relatively simple to use, requiring only the removal of the cap on the mouthpiece and a green button in the back to be pressed and released [38]. Successful inhalation of dose has occurred once the control window turns from the color green to red and an audible click is heard [38]. It was found in a randomized multicenter, crossover study that Genuair^®^ had a greater overall patient preference and satisfaction compared to the Breezhaler^®^ [47, 48]. Additionally, the number of patient attempts to result in a proper first inhalation was significantly less than with the Breezehaler^®^ [49]. The device offers an average FPF of 40%, a medium resistance with an average inspiratory rate of 64 L/min for effective drug de-agglomeration [41, 43]. The mean percentage of aclidinium bromide delivered in the airways and oropharynx of healthy adults was 30.1 ± 7.3% and 54.7 ± 7.2%, respectively [50].

The Ellipta^®^ is also a multidose DPI that was FDA-approved in 2013 [42]. It contains the corticosteroid fluticasone furoate combined with the LABA vilanterol [42]. Unlike the Genuair^®^, the Ellipta^®^ device requires even fewer steps in that the patient is only required to open the mouthpiece cover fully, inhale the powder, and close the mouthpiece [51]. The Ellipta^®^ device was found to have a 57% less incidence of handling errors with the first attempt compared to the Breezhaler^®^ [52]. In a questionnaire given to 1050 patients, 94% responded that the device was easy or very easy to use [53]. Ellipta^®^ is a medium resistance inhaler requiring an average inspiratory rate of 74 L/min [43]. However, it has been reported that the delivered doses of both the ICS and LABA in the Ellipta^®^ device are consistent over the flow range of 30–90 L/min [54]. Even patients with severe COPD could generate sufficient inspiratory flows for consistent drug delivery [55].

The Nexthaler^®^ is a multidose DPI containing a combination of the LABA formoterol fumarate and the corticosteroid beclomethasone dipropionate [32]. It was approved by the FDA in 2012 and is marketed as delivering extra-fine powder for the treatment of asthma [42]. Approximately one-third of the FPF is comprised of particles smaller than 1 micron [56]. Although particles this small do not get deposited in the oropharynx, they are likely to enter the bronchial tree and be exhaled [56]. It was found that 56% of the inhaled dose from a Nexthaler^®^ is delivered into the bronchial tree [57]. Additionally, the Nexthaler^®^ device is significantly easier to use compared to the Turbuhaler^®^ and the Diskus^®^ [58]. Like the Ellipta^®^, the Nexthaler^®^ is also relatively simple to use, requiring only a three-step process where the mouthpiece cover is opened fully, after which the patient may inhale the dose and close the cover [59]. It has medium–high resistance with inspiratory flow rates of around 54 L/min [43]. The FPF is between 40% and 45%, which is considered quite high for a DPI [41]. Nexthaler^®^ has consistent delivery of both drugs at flow rates between 30 and 90 L/min [60]

Unlike Genuair^®^, Ellipta^®^, and the Nexthaler^®^ devices, Breezhaler^®^ is a single-dose DPI that requires the loading of a drug-containing capsule prior to each inhalation [32]. It is indicated for the treatment of COPD and contains the LABA indacaterol as well as the LAMA glycopyrronium bromide [32]. It has been on the market longer than the other devices mentioned, with many studies using it as a comparator [47, 61]. The device requires the use of multiple steps and is associated with a greater number of errors [62]. It is characterized by a low intrinsic resistance with a minimum inspiratory flow of 50 L/min and an optimal range of 90–100 L/min [63, 64]. As a result of this device’s low resistance, it is appropriate for patients of varying disease severity [65]. It offers a lower resistance than the Handihaler^®^ as well as a 17% higher FPF and 14% lower oropharyngeal drug deposition [66, 67].

The quantity of active medication deposited in the airways with passive DPIs may have high patient variably due to differences in inspiratory flow rate [40]. Active DPIs utilize an external energy source, independent of patient inspiratory effort to de-agglomerate drug particles resulting in consistent drug delivery [39]. The Inspiromatic™ (Inspiro Medical, Misgav, Israel), a new active DPI undergoing phase 1 and 2 clinical trials, utilizes drug-filled capsules that are loaded into the device [68]. When the flow sensor detects patient inspiration, which can be a flow rate as low as 6 L/min, an active mechanism generates a pulsed vortexed flow inside the capsule which is then inhaled by the patient [68]. The device provides audio and visual feedback when the dose has been delivered completely and also records the time administered and overall patient inhaler performance in the memory chip; this data can then be downloaded and shared with the patient’s primary healthcare provider. Formoterol, delivered by Inspiromatic™ produced a statistically greater improvement in FEV_1_ at 15, 30, and 60 min compared to the Aerolizer^®^ [45, 68]. Another active DPI device that is still in development and has yet to undergo clinical trials is the Occoris^®^ (Team Consulting, Cambridge, UK). Occoris^®^ is an aerosolization engine that can be incorporated into various DPIs [45]. The company claims higher FPF and lower oropharyngeal drug deposition compared to typical passive DPIs [45].

## E-Technology in Asthma and COPD

The ability to use an inhaled device correctly plays a significant role in ensuring effective therapy [69]. Correct inhaler technique in the form of written instructions is insufficient and should include a practical demonstration as well as reminders and follow-up [70]. E-technology can provide a more consistent and standardized manner in educating patients about proper inhaler technique [71]. Additionally, these devices can provide key information to both physicians and patients to improve adherence [39]. This is important as 24% of asthma exacerbations and 60% of asthma-related hospitalizations are due to poor adherence [72]. Additionally, the use of e-technologies provides tools for patients to obtain general disease management education, self-monitor, obtain feedback, and identify trends and triggers [73]. Patient use of SABA was monitored in a study by Van Sickle et al. by attaching a device, known as Propeller, onto a pMDI [74]. The Propeller device, which is equipped with GPS, monitored the frequency, date, time, and location a SABA was used over 4 months. After the first month, patients began receiving weekly email reports of their SABA use. Interestingly, patients began having significant decreases in daytime and nighttime symptoms as well as an increased awareness and understanding of asthma level of control, triggers, and asthma patterns [75].

The VeriHaler (Sagentia, Cambridge, UK) is a device that is currently in development for the monitoring of inhaler adherence and performance [76]. The VeriHaler is a device comprising a microphone attached to the inhaler device casing. It is compatible with both pMDIs and DPIs. It utilizes an algorithm that removes unnecessary background noise and, on the basis of the acoustic signal detected, can sense the peak inspiratory flow rate, timing of inhalation compared to actuation of dose, and delivery of the formulation through the device. Feedback on inhaler performance is then sent via Bluetooth^®^ on an iPhone app where the user and their physician can discuss ways to improve inhaler performance or switch devices altogether. Another device in development is the T-Haler which is an MDI training device with the ability to monitor inhaler shaking, time of actuation, and inhalation flow [77]. It displays feedback to the user in the form of an interactive video game.

## Future of Asthma and COPD Treatment

### Personalized Medicine

In most asthma and COPD guidelines, treatment recommendations and algorithms usually follow a one size fits all approach [78]. Patients who do not respond to the highest doses of medications, despite being adherent, are labeled as having refractory illness [79]. Both airway diseases are highly heterogeneous in nature and present with different characteristics [80]. Genetic variability may lead to different immunologic mechanisms and responsiveness to medications [81]. This is important as understanding the genetic variability and mechanisms involved in the disease formation and progression can pave the way to the development of new personalized and targeted therapies [82]. For example, IL-33 is thought to play a role in airway remodeling in asthma; however, ICSs, which are the mainstream of therapy, do not inhibit its action [82]. Severe asthma may be divided into the Th2-high and Th2-low subtypes [83]. Patients with the Th2-high endotype are more likely to have increased airway hyerresponsiveness, high IL-4, IL-5, IL-13, and airway eosinophilia that is responsive to ICS [84]. In contrast, patients with Th2-low asthma tend to not be responsive to ICS, despite high doses [79]. It is in these patients that the high corticosteroid doses prescribed were inappropriate. There is already some literature exploring testing techniques in an attempt to identify these patients and hence avoid unnecessary drug exposure [85]. Th2-low patients may have high neutrophil counts and may benefit from a medication that reduces inflammation due to this [83]. Future treatments may also target specific cytokines present in Th2-high asthma as well as attempt to understand the mechanism of Th2-low disease to develop more personalized therapies.

In COPD, only select patients have an inflammatory phenotype, hence wide usage of ICS may provide no benefit with an increased risk of adverse effects [78]. After pooling ten randomized controlled trials, Pavord et al. found that there was a higher rate of pneumonia in patients with blood eosinophil counts less than 2% regardless of whether they were treated with an ICS or not [86]. Given the risks of acquiring a respiratory infection in those with severe COPD, future approaches may aim at avoiding unnecessary and prolonged use of ICS in patients with low eosinophil counts. Moreover, the cause and pathophysiologic processes that occur in a COPD exacerbation are heterogeneous [87]. Identifying biomarkers to have a clearer diagnosis of the type of exacerbation that occurred and how to prevent it could significantly decrease morbidity.

### Shared Decision-Making Between Patients and Physicians

Asthma and COPD are chronic diseases that require an ongoing patient–physician relationship. Today’s patients often desire a more active role in their healthcare as compared to the past [88]. Education around disease state, general approach to treatment, when to seek help, and action plans in the case of sudden worsening of symptoms enables patients to become more active in their care [12]. Patient participation in decision-making is known to lead to increased adherence rates, increased patient satisfaction, improved psychological adjustment to the condition, and better patient outcomes [89]. As part of the decision-making process, it is important to consider that inhaler devices used to treat asthma and COPD can be very expensive, and affordability issues are one of the top reasons for poor patient adherence [90]. Additionally, depending on the patient’s financial situation, affordability of product(s) may be the main factor that drives patient preference [91]. The wholesale costs of the most common inhalers used for asthma and COPD patients are listed in Table 3 [92]. Patient beliefs and values are important to consider in chronic disease management as patients who are skeptical of medication and the overall healthcare system may have poorer outcomes despite agreement on appropriate therapy [93]. Moreover, what constitutes good adherence may vary depending upon the patient’s personal views and culture [94]. Shared decision-making is a major component of patient-centered healthcare and involves participation of both patient and physician in all phases of care [95, 96]. A combination of evidence-based medicine and patient preference is more likely to lead to improvement in the health outcome the patient most values [97].

**Table 3:** Cost of some of the most common inhaler devices used in Canada

Trade name	Drug(s)	Device type	Doses per unit	Drug class	Cost per unit (CAD)^a,b^
Ventolin^®^	Salbutamol	MDI	200	SABA	6.50
Ventolin^®^	Salbutamol	Diskus^®^	60	SABA	12.56
Bricanyl^®^	Terbutaline	Turbuhaler^®^	100	SABA	8.65
Atrovent^®^	Ipratropium	MDI	200	SAMA	21.15
Flovent^®^	Fluticasone propionate	MDI	120	ICS	26.39 (50 mcg)
					45.51 (125 mcg)
					91.03 (250 mcg)
Flovent^®^	Fluticasone propionate	Diskus^®^	60	ICS	26.39 (100 mcg)
					45.53 (250 mcg)
					69.66 (500 mcg)
Pulmicort^®^	Budesonide	Turbuhaler^®^	200	ICS	33.93 (100 mcg)
					69.29 (200 mcg)
					100.91(400 mcg)
QVAR^®^	Beclomethasone	MDI	200	ICS	35.10 (50 mcg)
					70.00 (100 mcg)
Alvesco^®^	Ciclesonide	MDI	120	ICS	50.25 (100 mcg)
					83.07 (200 mcg)
Asmanex^®^	Mometasone	Twisthaler^®^	120	ICS	69.29 (200 mcg)
					100.91(400 mcg)
Serevent^®^	Salmeterol	Diskus^®^	60	LABA	63.73
Onbrez^®^	Indacaterol	Breezhaler^®^	30	LABA	50.46
Advair^®^	Salmeterol + fluticasone propionate	Diskus^®^	60	LABA + ICS	89.77 (100 mcg)
					107.46 (250 mcg)
					152.55 (500 mcg)
Advair^®^	Salmeterol + fluticasone propionate	MDI	120	LABA + ICS	107.46 (125 mcg)
					152.55 (250 mcg)
Symbicort^®^	Formoterol + budesonide	Turbuhaler^®^	120	LABA + ICS	72.50 (100 mcg)
					94.22 (200 mcg)
Zenhale^®^	Formoterol + mometasone	MDI	120	LABA + ICS	96.04 (100 mcg)
					116.40 (200 mcg)
Breo^®^	Vilanterol + fluticasone furoate	Ellipta^®^	30	LABA + ICS	89.19 (100 mcg)
					139.69 (200 mcg)
Spiriva^®^	Tiotropium	Handihaler^®^	30	LAMA	56.32
Spiriva^®^	Tiotropium	Respimat^®^	30	LAMA	56.32
Seebri^®^	Glycopyrronium	Breezhaler^®^	30	LAMA	57.62
Tudorza^®^	Aclidinium	Genuair^®^	60	LAMA	57.62
Incruse^®^	Umeclidinium	Ellipta^®^	30	LAMA	54.25
Duaklir^®^	Aclidinium + formoterol	Genuair^®^	60	LAMA + LABA	65.10
Anoro^®^	Umeclidinium + vilantero	Ellipta^®^	30	LAMA + LABA	87.89
Ultibro^®^	Glycopyrronium + indacterol	Breezhaler^®^	30	LAMA + LABA	87.24
Inspiolto^®^	Tiotropium + olodaterol	Respimat^®^	60	LAMA + LABA	66.08

Currency conversion may be used to estimate cost in other countries

*SABA* short-acting β_2_-agonist, *SAMA* short-acting antimuscarinic antagonist, *LABA* long-acting β_2_-agonist, *LAMA* short-acting antimuscarinic antagonist, *ICS* inhaled corticosteroid

^a^Prices are wholesale costs obtained from McKesson Canada June 2017 excluding pharmacy markup and dispensing fee [92]

^b^In combination products, the strength listed in the cost column reflects the ICS dose only

## Conclusions

Asthma and COPD are both chronic respiratory conditions that usually require long-term treatment. As with many chronic health conditions, asthmatics and COPD patients have a relatively low adherence rate. Several relatively new inhalers as well as electronic devices designed to improve patient adherence have become commercially available. With the variety of medications and devices available, it is important for physicians to allow not only evidence-based medicine but also patient preference to guide the development of the therapeutic plan.
